# Contrast clearance analysis in post-operative brain metastases resection cavities: case series and literature review

**DOI:** 10.3389/fonc.2026.1903810

**Published:** 2026-09-04

**Authors:** Anna Pokrzywa, Maciej Blok, Magdalena Adamczak-Sobczak, Szymon Ziółkowski, Maciej Harat

**Affiliations:** 1 Department of Neurooncology and Radiosurgery, Franciszek Lukaszczyk Memorial Oncology Center, Bydgoszcz, Poland; 2 Department of Clinical Medicine, Jan and Jedrzej Sniadecki University of Science, and Technology, Bydgoszcz, Poland; 3 Department of Oncology, Nicolaus Copernicus University, Ludwik Rydygier Collegium Medicum, Bydgoszcz, Poland

**Keywords:** brain metastasis, contrast clearance analysis, imaging appearances, post-operative cavity, TRAMs

## Abstract

**Background:**

The aims of this study were to assess how previously described treatment response assessment maps/contrast clearance analysis (TRAMs/CCA) imaging appearances may present in post-operative brain metastasis resection cavities and to discuss their potential contribution to clinical decision-making.

**Methods:**

This retrospective descriptive case series included four patients with surgically resected brain metastases. Cases were purposively selected to illustrate distinct post-operative TRAMs/CCA imaging appearances resembling those previously described after stereotactic radiosurgery (SRS). All included patients had histologically confirmed metastatic disease and underwent routine post-operative magnetic resonance imaging (MRI) surveillance. TRAMs/CCA imaging was performed using a 1.5-T MRI scanner according to the Brainlab Elements protocol for CCA with standard-dose gadolinium administration. Early and delayed post-contrast 3D T1-weighted sequences were analyzed to generate contrast clearance maps, providing complementary imaging information regarding contrast enhancement characteristics of post-operative lesions. Post-operative cavities were assessed in multidisciplinary consensus.

**Results:**

Four representative CCA/TRAMs imaging appearances within post-operative resection cavities, showing features analogous to TRAMs/CCA imaging features previously described after SRS, were observed and correlated with conventional contrast-enhanced T1-weighted MRI findings and follow-up outcomes. Appearances characterized by the absence of early contrast clearance or by extensive persistent delayed retention despite marked contrast enhancement were observed in cases with post-operative changes without local recurrence; in these cases, adjuvant SRS was deferred and MRI surveillance was performed. Mixed TRAMs/CCA appearances with focal areas of early contrast clearance were observed in a case that subsequently developed local recurrence within regions considered suspicious on TRAMs/CCA. In contrast, appearance characterized by a thin peripheral early contrast clearance with adjacent delayed contrast retention was observed in a case with intermediate post-operative findings for which follow-up imaging was not available.

**Conclusions:**

TRAMs/CCA may represent a useful non-invasive adjunct to conventional MRI for SRS planning after brain metastases resection. These findings support further investigation of TRAMs/CCA as a potential adjunct for treatment planning and longitudinal surveillance. Further prospective studies are warranted.

## 1 Introduction

Differentiating various post-operative changes following resection of brain metastases (BMs) presents a significant challenge in planning radiotherapy to the post-operative cavity. The extent of surgical resection is usually assessed by magnetic resonance imaging (MRI) within 48 h of surgery (1). At this early time point, scar tissue or smear enhancement has not yet formed, facilitating differentiation from residual tumor mass (2). However, a lack of timely post-operative MRI or disease progression between surgery and stereotactic radiosurgery (SRS) often makes it difficult to accurately define the optimal target for SRS (3). Post-contrast enhancement may represent post-operative scarring, potentially leading to an unnecessarily enlarged irradiated volume and increased risk of radiation necrosis, which has been reported in 5%–50% of cases across various studies (4–8).

Assessment of post-operative residual tumor volume is also an important factor in planning adjuvant SRS after BM resection. Consequently, accurate post-operative imaging assessment is essential not only to document the extent of resection but also to support decisions regarding adjuvant therapies, particularly SRS (9, 10).

Conversely, a failure to treat residual tumor may risk local recurrence, which occurs in 10%–50% of cases after SRS (11–13). Therefore, to significantly improve the safety and efficacy of radiosurgery, it is crucial to implement simple and readily available diagnostic methods in radiotherapy treatment planning that help distinguish between scar tissue and tumor recurrence (14, 15).

Of particular interest are methods that may constitute a promising complementary tool and can be implemented and easily analyzed by radiation oncologists, and that do not require the involvement of external factors or the use of medical resources in busy oncology facilities. Such an auxiliary tool may be treatment response assessment maps/contrast clearance analysis (TRAMs/CCA). The practical implementation of TRAMs/CCA has recently offered new opportunities for monitoring patients with brain tumors after radiotherapy (16). Although advanced MRI techniques such as diffusion-weighted imaging (DWI) and perfusion-weighted imaging (PWI) provide complementary information to conventional MRI, their interpretation in the post-operative setting remains challenging because of overlapping imaging features between residual tumor and post-operative treatment-related changes. Furthermore, variability in acquisition protocols and image analysis limits standardization across institutions. In this context, TRAMs/CCA may represent an additional imaging technique that provides complementary information to conventional and advanced MRI in the assessment of post-operative cavity changes. While the clinical utility of TRAMs/CCA has already been described in the evaluation of treatment response following radiotherapy, particularly for differentiating viable tumor from treatment-related changes, its role in the post-operative assessment of BM resection cavities has not yet been established (15, 17, 18).

The TRAMs/CCA method is based on acquiring two standard 3D T1-weighted MRI sequences, one performed 5 min and the other 60–105 min after administration of a gadolinium-based contrast. The delayed image is subtracted from the early one, and the result is analyzed to generate volumetric maps distinguishing regions of rapid contrast clearance (blue) from regions of contrast accumulation (red) (18). The contrast is cleared more quickly from tumor tissue due to its rich and leaky vasculature, while it accumulates slowly in fibrotic scar tissue.

Although TRAMs/CCA imaging appearances have been described mainly in the assessment of treatment-related changes after BM radiotherapy, their application to the post-operative evaluation of BMs resection cavities, in our knowledge, has not been reported. Whether these established imaging appearances can also be recognized in this setting and provide clinically relevant information remains unknown (15).

The aim of this study was to assess how previously described TRAMs/CCA imaging appearances may present in post-operative BM resection cavities and to discuss their potential contribution to clinical decision-making.

## 2 Methods

This was a retrospective descriptive case series conducted at the Department of Neuro-oncology and Radiosurgery, Oncology Center in Bydgoszcz, Poland. We included four patients with histologically confirmed BMs who underwent surgical resection and standard post-operative MRI surveillance. All patients were initially considered for adjuvant radiotherapy; however, further treatment was deferred following multidisciplinary review incorporating conventional MRI and TRAMs/CCA findings. Cases were purposively selected for detailed analysis based on distinct post-operative CCA/TRAMs imaging appearances. The selection was intended to illustrate imaging findings resembling those previously reported after SRS and to explore their potential relevance in multidisciplinary clinical decision-making. This study was descriptive in nature and was not designed to assess the diagnostic accuracy, predictive performance, or clinical utility of CCA/TRAMs.

All patients underwent TRAMs/CCA imaging as part of their standard post-operative follow-up using a 1.5-T MRI scanner (Siemens Healthineers Erlangen Germany) according to the Brainlab Elements protocol for CCA. The protocol included the administration of a standard intravenous dose of a gadolinium-based contrast agent (0.1 mmol/kg). Two high-resolution 3D T1-weighted gradient-echo sequences were acquired 3–5 min after contrast administration and 60 ± 10 min after contrast administration. Repetition time (TR), echo time (TE), field of view (FOV), and slice thickness were kept constant between the early and delayed phases to ensure accurate subtraction and registration.

Post-processing was performed using the Brainlab Elements Contrast Clearance Analysis module Brainlab Elements Multiple Brain Mets SRS, version 4.5 (Brainlab AG, Munich, Germany). The delayed T1 sequence was rigidly registered to the early T1 sequence, and voxel-wise subtraction was used to create parametric maps of contrast kinetics. Voxels with rapid contrast clearance were visualized in blue and were interpreted as consistent with viable tumor due to active perfusion and blood–brain barrier disruption. Voxels with delayed contrast retention were marked red and were interpreted as treatment-related effects such as fibrosis, necrosis, or post-surgical change. Regions of interest (ROIs) were drawn manually or semi-automatically around the post-operative cavity by an experienced neuroradiologist and reviewed by a radiation oncologist. CCA maps were interpreted in consensus by a multidisciplinary team of neuro-oncologists, radiation oncologists, and neuroradiologists. The imaging appearances described in this study were adapted from previously reported TRAMs/CCA features observed after radiotherapy. Their application to the post-operative assessment of BMs resection cavities has not been systematically investigated and represents the focus of the present study.

All patients provided written informed consent for data collection before treatment as part of the Central Nervous System Cancer Registry at the Oncology Center in Bydgoszcz, Poland. The Bioethics Committee of Nicolaus Copernicus University in Torun, Collegium Medicum in Bydgoszcz, Poland approved the study (KB720/2018). All methods were conducted in accordance with the Declaration of Helsinki and institutional ethical standards.

## 3 Results

**Figure 1:**
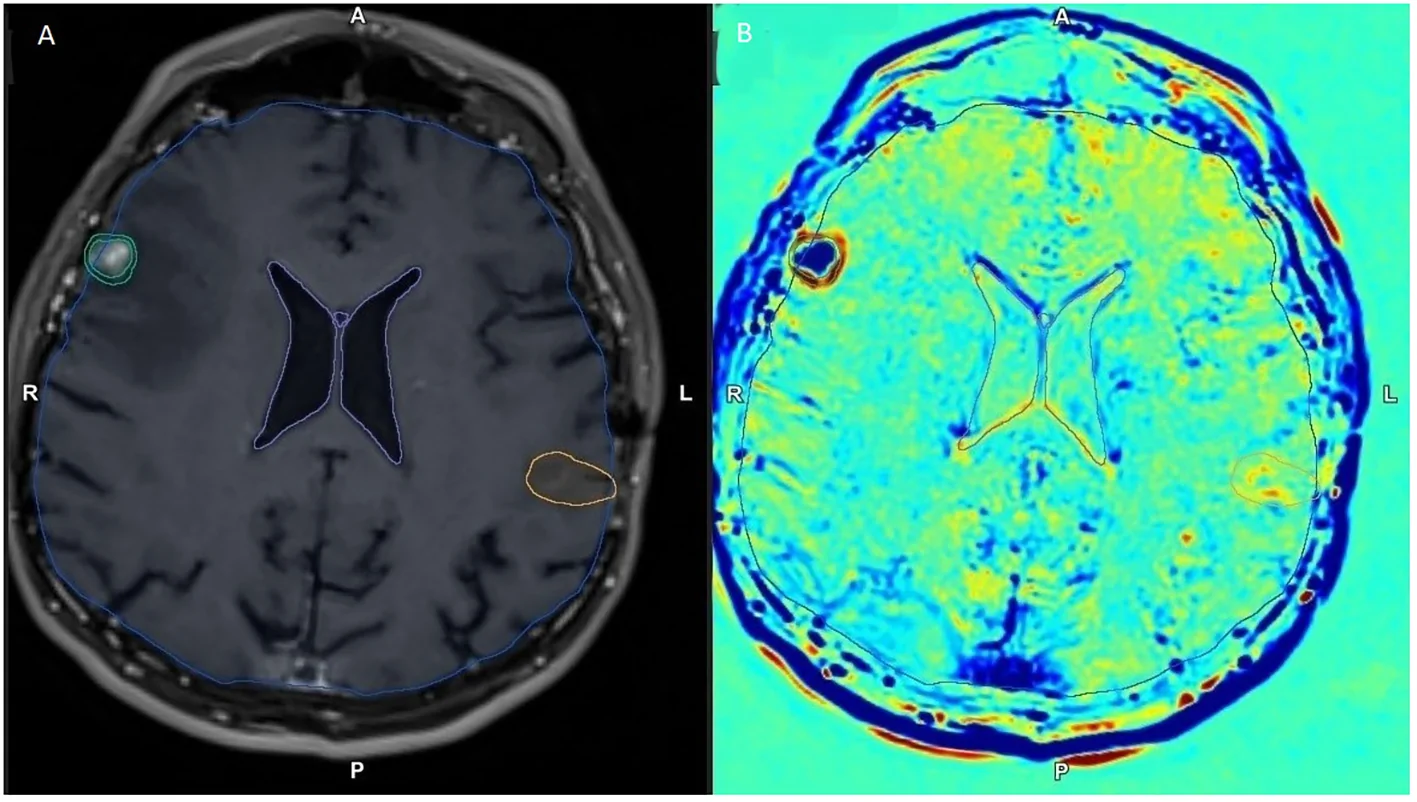
Post-operative TRAMs/CCA findings demonstrating the absence of early contrast clearance and delayed contrast retention within the resection cavity (area marked in orange). **(A)** Contrast-enhanced T1-weighted MRI acquired for treatment planning showing post-operative enhancement of the resection cavity. **(B)** TRAMs/CCA demonstrating the absence of early contrast clearance and delayed contrast retention 8 months post-surgery. Additional active tumor identified in the right frontal lobe (area marked in green).

**Figure 2:**
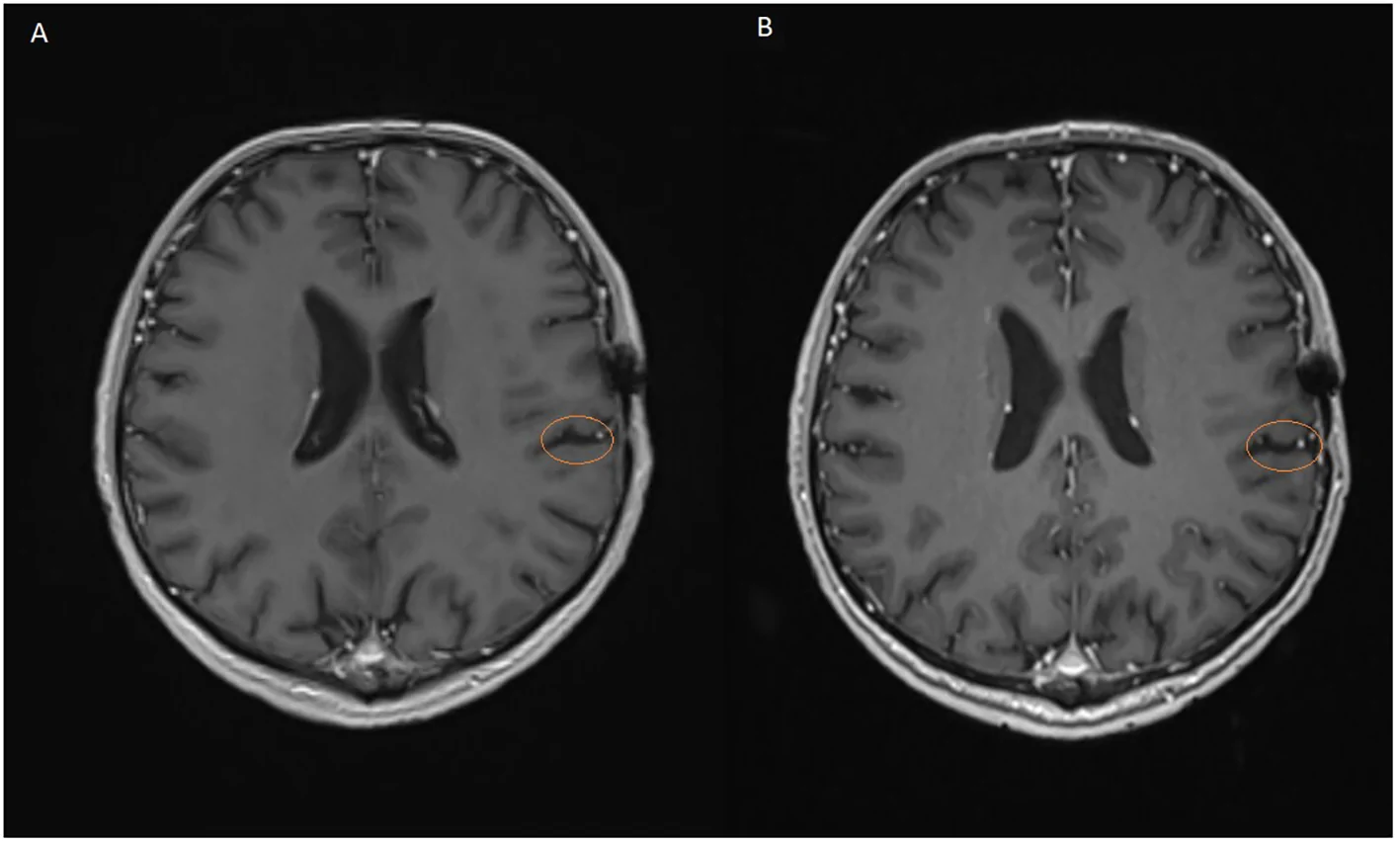
Follow-up contrast-enhanced T1-weighted MRI at 12 months **(A)** and 15 months **(B)** post-surgery showing stable post-operative changes without evidence of local recurrence (area marked in orange).

Four selected cases demonstrating post-operative TRAMs/CCA imaging findings after BM resection, similar to those previously described after SRS, are presented. These findings were evaluated in relation to fat-suppressed contrast-enhanced T1-weighted MRI acquired for treatment planning and, where available, subsequent follow-up imaging. An overview of the four clinical cases, including conventional post-operative MRI findings, additional information provided by TRAMs/CCA, clinical decisions based on the overall clinical and imaging assessment, and follow-up outcomes, is presented in **Table 1**. MRI for SRS planning undertaken 8 months after resection showed enhancement suggestive of recurrence (**Figure 1A**). However, TRAMs/CCA analysis demonstrated absence of both early contrast clearance and delayed contrast retention within the resection cavity (**Figure 1B**). No local recurrence was observed during follow-up. Based on the overall clinical and imaging assessment, adjuvant radiotherapy was deferred. At follow-up MRI performed 12 (**Figure 2A**) and 15 (**Figure 2B**) months after surgery, no contrast enhancement was observed within the resection cavity.

**Table 1:** Summary of conventional post-operative MRI findings, additional information provided by CCA/TRAMs, clinical management decision, and follow-up outcome.

Case	Conventional post-operative MRI findings	Additional information provided by TRAMs/CCA	Clinical decision based on overall clinical and imaging assessment	Follow-up outcome
1	Contrast enhancement within the resection cavity, suspicious for recurrence	Absence of both early contrast clearance and delayed contrast retention within the resection cavity	Adjuvant SRS was deferred, MRI surveillance	No local recurrence
2	Extensive contrast enhancement along the resection cavity margins, suspicious for recurrence	Persistent delayed retention without early contrast clearance	Adjuvant SRS was deferred, close clinical and MRI surveillance	No local recurrence
3	Equivocal enhancement at the resection cavity margin	Focal contrast clearance adjacent to delay contrast retention	Adjuvant SRS was deferred, close clinical and MRI surveillance	Local recurrence
4	Heterogenous signal intensity within the resection cavity and equivocal marginal enhancement	Thin peripheral rim of contrast clearance adjacent to delay contrast retention	Adjuvant SRS was deferred, longitudinal MRI surveillance planned	Follow-up not available

### Case 1: absence of early contrast clearance and delayed contrast retention

A 57-year-old man with metastatic lung adenocarcinoma to the left parietal lobe had prior SRS in 2023 and surgery in 2024. The patient was treated with docetaxel and nivolumab. A first follow-up MRI was performed 2 months after surgery, showing post-operative changes. MRI for SRS planning undertaken 8 months after resection showed enhancement suggestive of recurrence. However, TRAMs/CCA analysis demonstrated the absence of both early contrast clearance and delayed contrast retention within the resection cavity. No local recurrence was observed during follow-up. Based on the overall clinical and imaging assessment, adjuvant radiotherapy was deferred. At follow-up MRI performed 12 months after surgery, no contrast enhancement was observed within resection cavity. TRAMs/CCA analysis was not repeated.

This case demonstrated TRAMs/CCA findings where neither early contrast clearance nor delayed retention is present within the resection cavity, resembling previously described appearances in the post-SRS treatment. Subsequent imaging confirmed stable post-operative findings without evidence of local recurrence.

### Case 2: persistent delayed contrast retention without early contrast clearance

**Figure 3:**
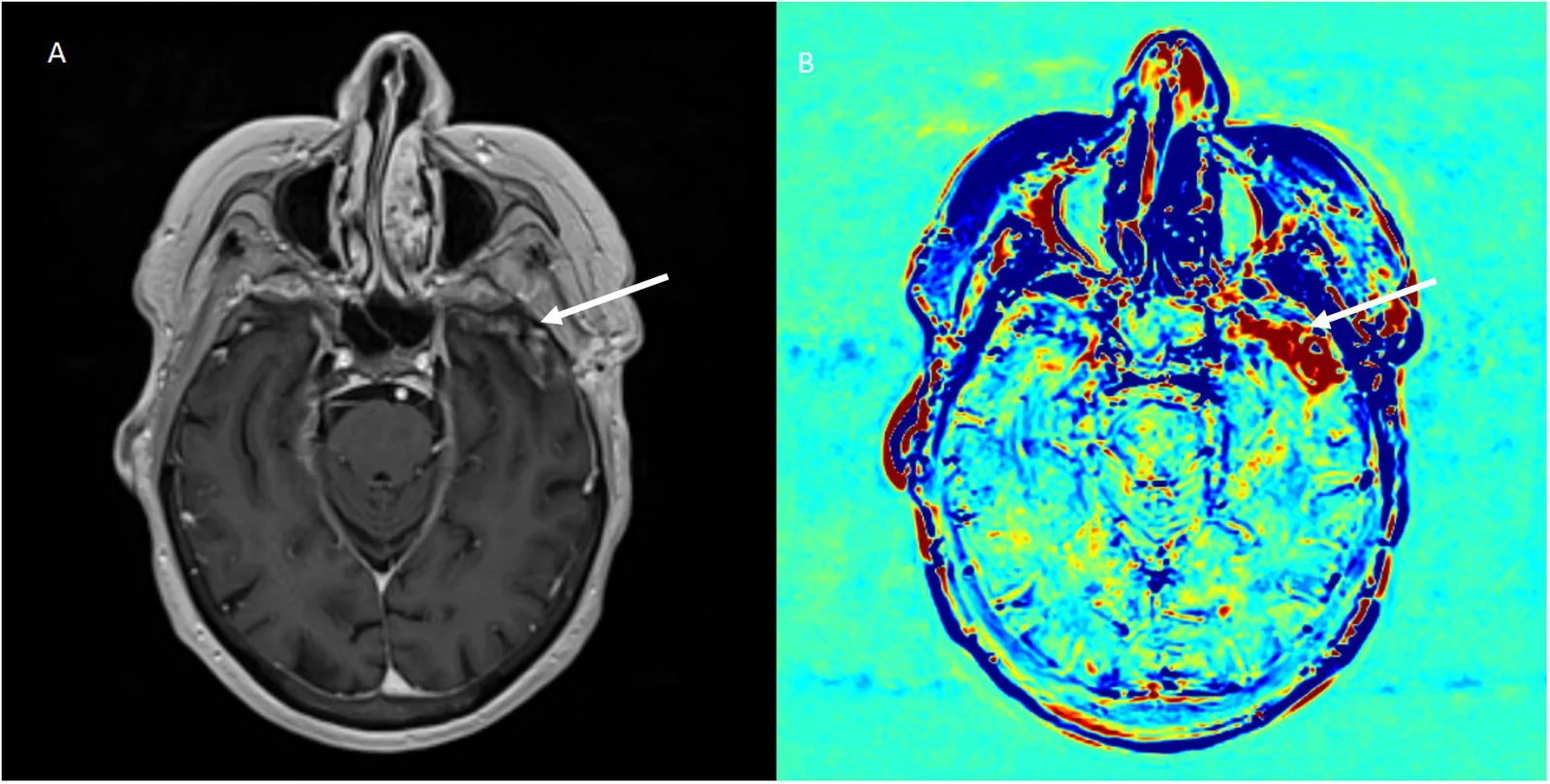
Post-operative TRAMs/CCA findings demonstrating persistent delayed contrast retention without early contrast clearance within the contrast enhancement on the edge of the resection cavity. **(A)** Contrast-enhanced T1-weighted MRI acquired for SRS planning 2 months after resection demonstrating extensive post-operative contrast enhancement. **(B)** TRAMs/CCA demonstrating persistent delay contrast retention without early contrast clearance.

**Figure 4:**
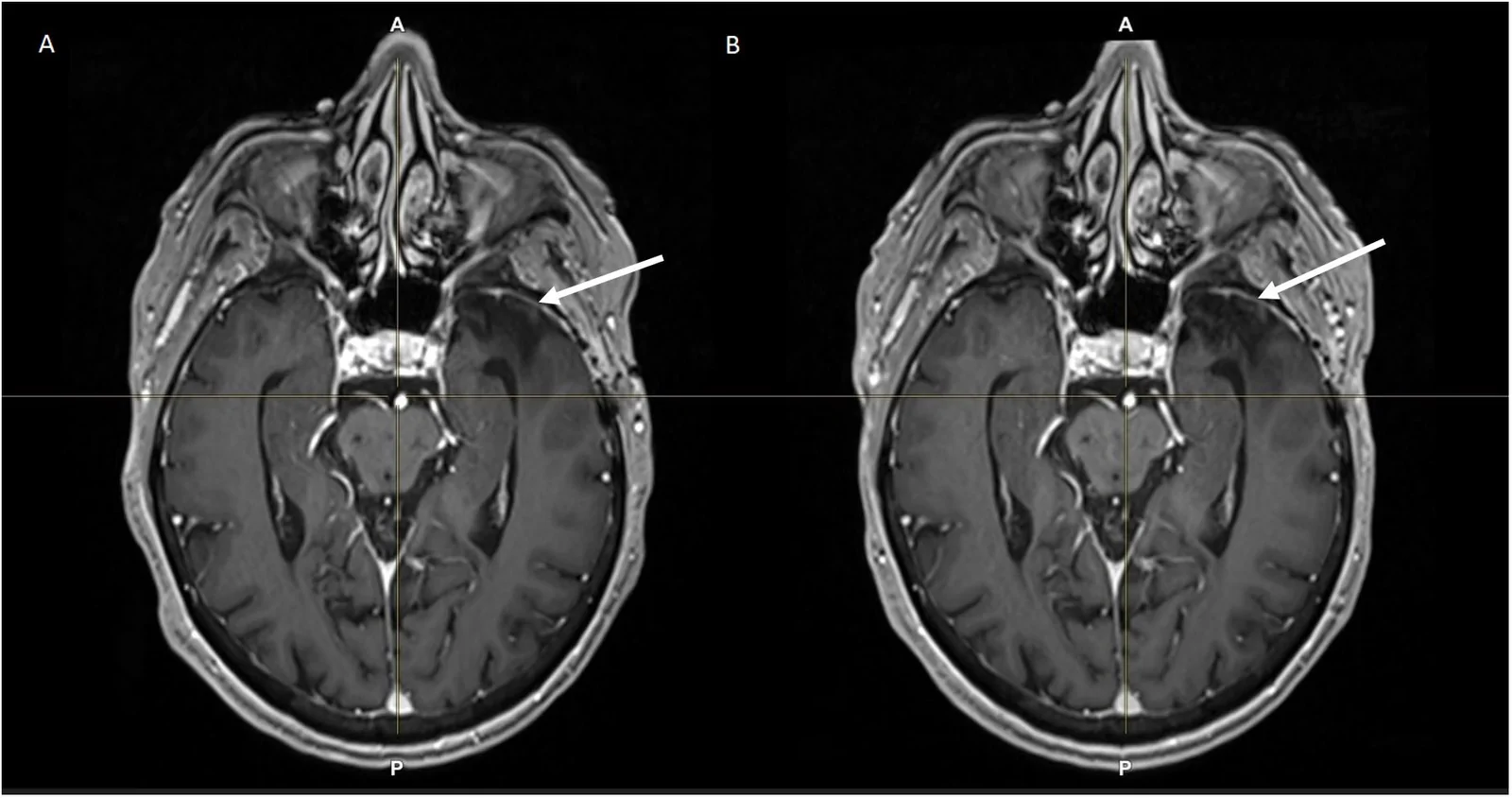
Follow-up contrast-enhanced T1-weighted MRI at 6 months **(A)** and 9 months **(B)** post-surgery showing post-operative changes without evidence of local recurrence.

An 86-year-old woman with a history of melanoma BM to the left temporal region underwent SRS in 2020, reirradiation in 2023, and surgical resection in 2025 due to lesion progression. Histopathology confirmed recurrent tumor. A computed tomography (CT) scan performed within 48 h of surgery revealed a post-operative cavity without any detectable residual tumor. MRI performed for SRS planning undertaken 2 months after resection demonstrated extensive enhancement suggestive of tumor recurrence (**Figure 3A**). TRAMs/CCA showed persistent delayed contrast retention without early contrast clearance (**Figure 3B**). Considering the overall clinical and imaging findings, the patient underwent clinical and MRI surveillance, and further treatment was deferred. Follow-up MRI at 6 (**Figure 4A**) and 9 (**Figure 4B**) months after treatment demonstrated post-resection stable cavity without evidence of local recurrence.

This case demonstrated TRAMs/CCA findings characterized by persistent delayed retention without early contrast clearance within the contrast enhancement on the edge of the resection cavity. These imaging findings resembled previously described TRAMs/CCA appearances associated with post-SRS treatment. Subsequent imaging confirmed stable post-operative findings without evidence of local recurrence.

### Case 3: focal contrast clearance with adjacent delayed contrast retention

**Figure 5:**
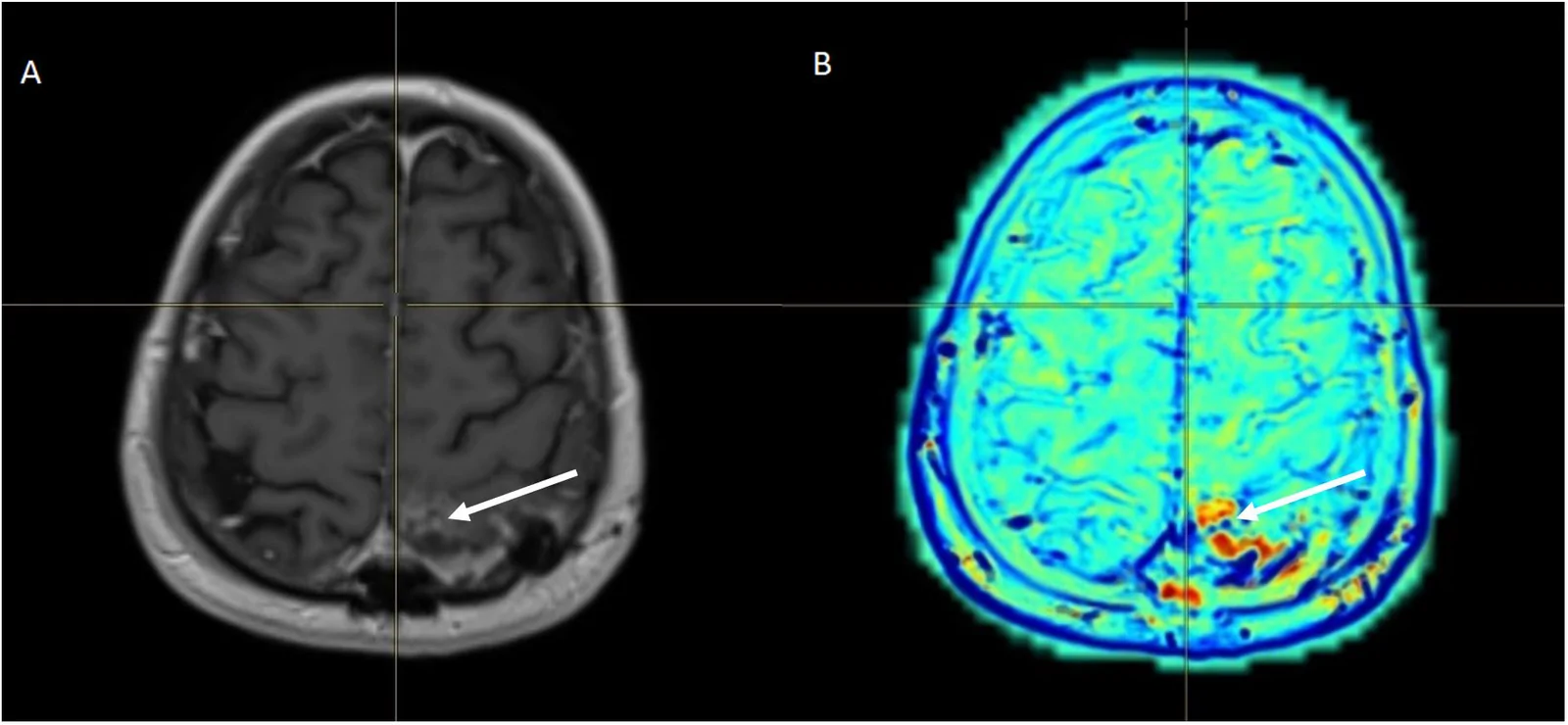
Post-operative TRAMs/CCA findings demonstrating focal contrast clearance adjacent to areas of delay contrast retention with the contrast-enhancing tissue surrounding the resection cavity. **(A)** Contrast-enhanced T1-weighted MRI performed 25 days after resection for SRS planning demonstrating equivocal post-operative contrast enhancement. **(B)** TRAMs/CCA demonstrating focal contrast clearance adjacent to areas of delayed contrast retention.

**Figure 6:**
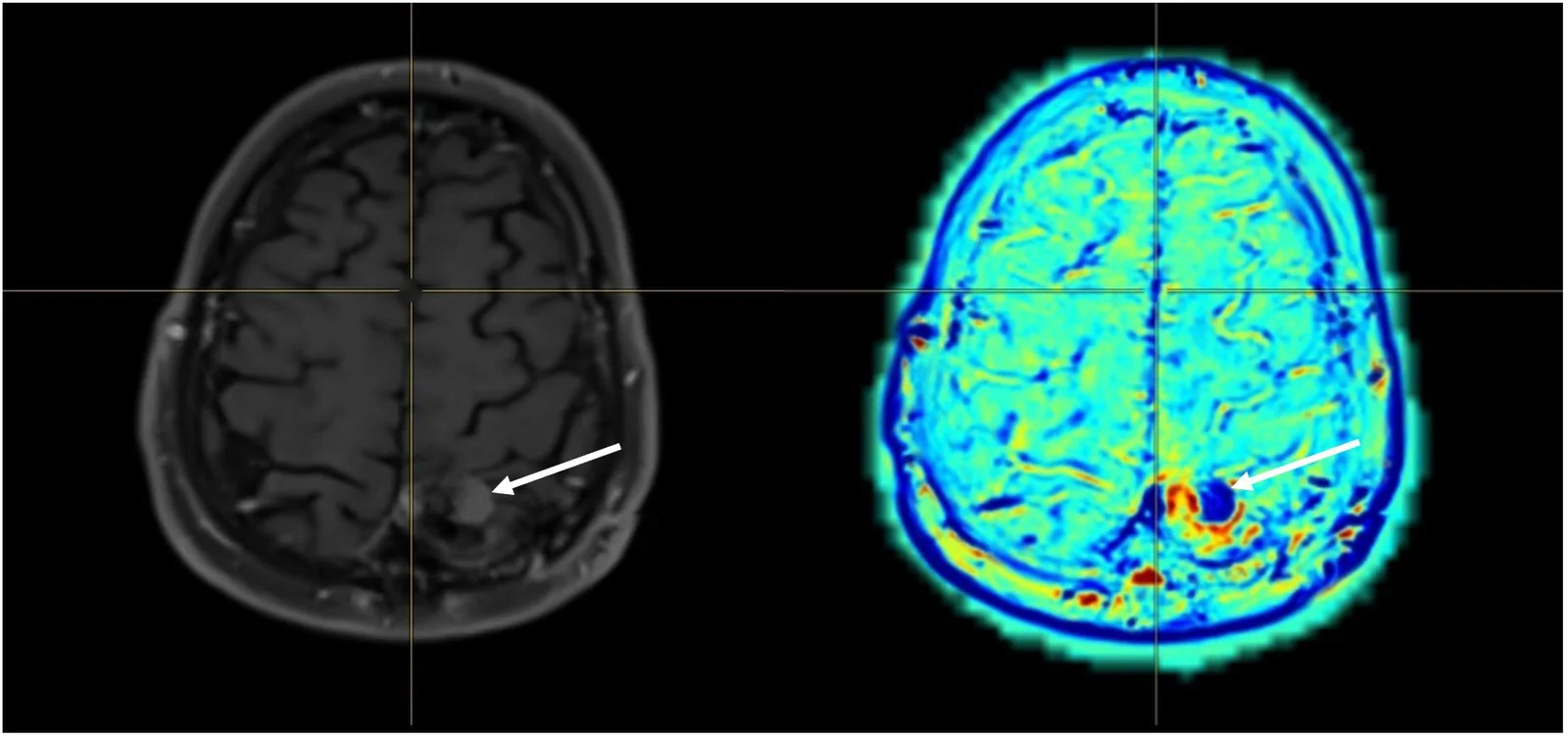
**(A)** Follow-up contrast-enhanced T1-weighted MRI at 7 months after surgery demonstrating local recurrence. **(B)** Corresponding to follow-up TRAMs/CCA confirming contrast clearance within the recurrent lesion.

A 52-year-old man with metastatic breast cancer to the left frontoparietal area underwent complete surgical resection in 2025. The patient was treated systemically with aromatase inhibitors, CDK4/6 inhibitors, and LHRH analogs. MRI performed within 48 h of surgery revealed post-operative changes, without evidence of residual enhancing tumor. MRI performed 25 days later for SRS planning showed equivocal contrast enhancement (**Figure 5A**). TRAMs/CCA demonstrated focal contrast clearance adjacent to areas of delay contrast retention within the enhancing tissue (**Figure 5B**). Based on the overall clinical and imaging assessment, the patient was managed with close clinical and MRI surveillance. Follow-up MRI (**Figure 6A**) and TRAMs/CCA (**Figure 6B**) performed 7 months after surgery demonstrated local recurrence within the region corresponding to the previously identified focal contrast clearance.

This case demonstrated TRAMs/CCA findings characterized by focal contrast clearance surrounded by contrast retention. Similar heterogeneous TRAMs/CCA imaging appearances have been described in the post-SRS treatment. In this patient, subsequent imaging demonstrated local recurrence within the region corresponding to the previously identified focal contrast clearance, while the surrounding post-operative changes remained stable and were not included in the SRS target volume.

### Case 4: peripheral focal contrast clearance with adjacent delayed contrast retention

**Figure 7:**
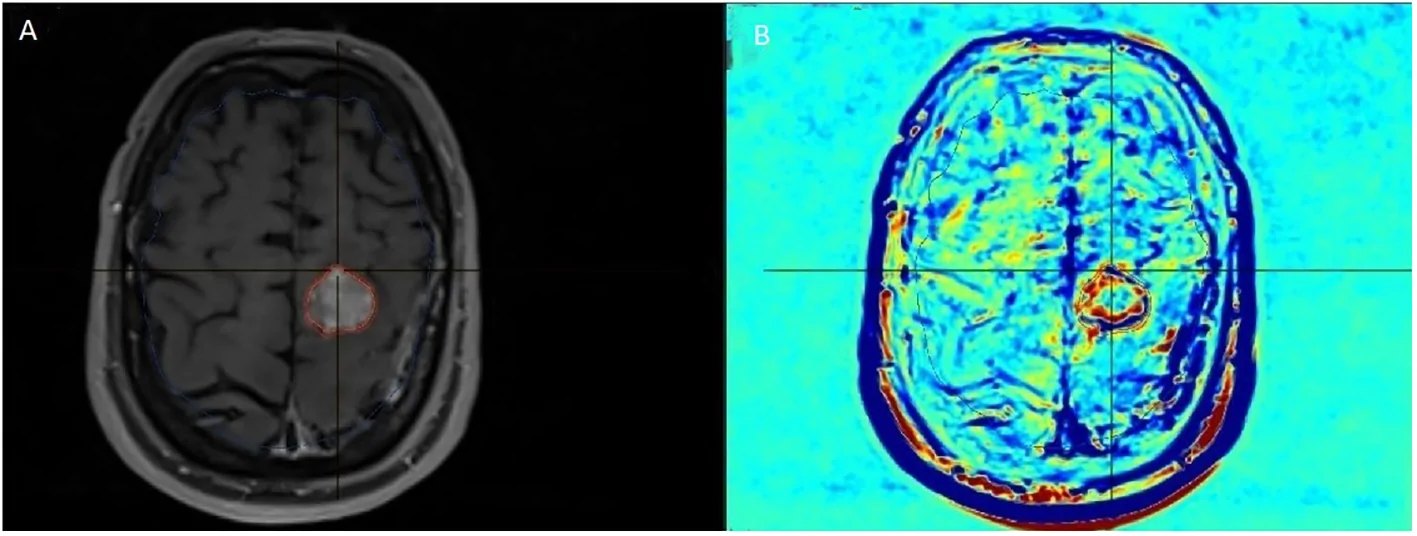
Post-operative TRAMs/CCA findings demonstrating a thin peripheral rim of contrast clearance adjacent to areas of delayed contrast retention surrounding the resection cavity. **(A)** Contrast-enhanced T1-weighted MRI performed 28 days after resection for SRS planning demonstrating heterogeneous signal intensity within the resection cavity and equivocal marginal enhancement. **(B)** Corresponding TRAMs/CCA demonstrating a central area without significant contrast clearance or delayed contrast retention, surrounded by delayed contrast retention and a thin peripheral rim of early contrast clearance.

A 69-year-old man with metastatic colon adenocarcinoma to the left frontal lobe underwent complete surgical resection in 2025. MRI for SRS planning performed 28 days after resection demonstrated heterogeneous signal intensity within the resection cavity and equivocal marginal enhancement (**Figure 7A**). TRAMs/CCA demonstrated a focal peripheral area of contrast clearance corresponding to a thin peripheral rim, with adjacent areas of delayed contrast retention surrounding the resection cavity (**Figure 7B**). The central cavity showed no significant areas of either contrast clearance or delayed contrast retention. Based on these imaging findings, the peripheral contrast clearance was considered suspicious for possible residual tumor at the cavity margin, although no follow-up confirmation is currently available.

This case demonstrated TRAMs/CCA findings in which the central cavity shows no significant contrast retention or clearance, while a surrounding area exhibits delayed contrast retention, which itself is encircled by a thin peripheral rim of early contrast clearance—a thin blue rim. This appearance has been associated with residual or recurrent tumor at the cavity margin as the central neutral zone and the intermediate area of delayed retention likely represent post-surgery changes, whereas the peripheral wash-out reflects tumor at the edge of the resection cavity.

## 4 Discussion

Radiation necrosis and scar tissue formation in the tumor bed are common effects of radiotherapy and surgery, respectively. Both can develop new contrast enhancements after contrast agent administration and show similar imaging features to tumor progression on standard MRI (14, 15). Understanding the dynamics of changes occurring in the post-operative bed is important for optimizing the timing of further treatment and for accurate identification of patients who may benefit from adjuvant irradiation while avoiding unnecessary treatment. While timing and outcomes of radiotherapy after surgery have been evaluated for glioblastoma, similar data for metastasis are lacking (3, 19). Biopsy is currently the best diagnostic tool for recurrence, but it is invasive and carries the risk of incorrect sampling due to the heterogeneity of necrotic tumors (20). Current imaging methods have their own limitations; for example, MRS reveals metabolic changes in brain tissue that can be helpful in distinguishing between recurrent tumor and radiation necrosis (12), but it can be difficult to interpret and lacks specificity. To minimize the number of unnecessary medical interventions and related complications, effective diagnostic methods are needed to differentiate post-treatment changes from persistent neoplastic tissue. TRAMs/CCA has emerged as a promising adjunct to conventional MRI for differentiating treatment-related changes from tumor progression. Histopathological correlation reported in previous studies has shown that areas of early contrast clearance are more frequently associated with viable tumor, whereas delayed contrast retention is more commonly observed in treatment-related changes (18, 21). Retrospective studies have demonstrated encouraging diagnostic performance of TRAMs/CCA, with higher sensitivity reported compared with conventional MRI and diagnostic performance comparable to that of perfusion-weighted MRI, suggesting a potential complementary role alongside advanced MRI techniques (15, 20). CCA can be readily incorporated into standard MRI protocols because it does not require hardware or radiotracers and provides high-resolution volumetric information. Nevertheless, it is limited by reduced accuracy in detecting very small lesions (<5 mm), dependence on contrast agent uptake, and the potential for false positives in the context of antiangiogenic therapy or atypical vascular responses (16, 20). Despite evidence supporting post-operative SRS for improving local control, selecting patients who require immediate adjuvant treatment remains challenging, particularly when post-operative enhancement cannot be confidently distinguished from treatment-related or surgical changes on conventional MRI. Previous studies have shown that post-operative SRS reduces local recurrence compared with observation alone, although the benefit depends on multiple factors, including cavity size and extent of resection (22).

Several characteristic CCA/TRAMs imaging appearances have been described in the literature based on the presence and spatial distribution of early contrast clearance and delayed contrast retention, reflecting different contrast kinetics following radiotherapy, particularly SRS. Areas of early contrast clearance have been associated with viable tumor, whereas delayed contrast retention has been more commonly associated with treatment-related changes. Mixed imaging appearances may be more difficult to interpret and often require correlation with the clinical context and additional imaging findings. For example, focal contrast clearance surrounded by delayed contrast retention has been reported in lesions containing both viable tumor and treatment-related changes, whereas delayed contrast retention encircled by a thin peripheral rim of early contrast clearance has been described in association with central treatment-related changes and possible residual tumor at the lesion margin (18).

To our knowledge, this is among the first reports describing CCA/TRAMs imaging appearances in post-operative BM resection cavities before adjuvant radiotherapy. Rather than evaluating the diagnostic accuracy of TRAMs/CCA, we describe four representative post-operative imaging appearances and discuss their potential contribution to multidisciplinary clinical decision-making when interpreted alongside conventional MRI findings and clinical follow-up.

In the present series, imaging appearances characterized by the absence of early contrast clearance or predominant delayed contrast retention were associated with stable post-operative findings during follow-up, whereas focal areas of early contrast clearance were observed in a case that subsequently developed local recurrence. Another case demonstrated a peripheral rim of early contrast clearance with adjacent delayed contrast retention, representing an indeterminate post-operative appearance for which follow-up imaging was unavailable. Although these observations cannot establish diagnostic accuracy, they illustrate how TRAMs/CCA may provide complementary information alongside conventional MRI in selected post-operative scenarios. Current RANO recommendations acknowledge the potential value of advanced imaging techniques, including diffusion- and perfusion-weighted MRI, but emphasize that insufficient standardization currently limits their incorporation into formal response assessment criteria. In this context, TRAMs/CCA may represent an additional source of complementary imaging information, although further validation is required before broader clinical implementation (23).

This study has several limitations that should be considered. The main limitation is the small sample size of four patients, which precludes statistical analysis and limits the generalizability of the findings. Another limitation is the intentional selection of cases from a larger cohort because they illustrated imaging appearances relevant to the objectives of this report, introducing an inherent selection bias. In addition, follow-up imaging was unavailable for Case 4, as the patient underwent subsequent imaging at another institution and the results were not accessible.

The cohort was also heterogeneous with respect to primary tumor histology, patient age, systemic therapy, follow-up duration, and previous treatment history, including preoperative radiotherapy in some patients. Although this heterogeneity limits direct comparison between individual cases, it reflects the diversity of patients encountered in routine clinical practice.

The purpose of this report was not to evaluate the diagnostic accuracy of CCA/TRAMs or estimate the frequency of specific imaging appearances, but rather to illustrate clinically relevant post-operative scenarios in which CCA/TRAMs may provide complementary information alongside conventional MRI. Our observations suggest that imaging appearances previously described after SRS may also be encountered in post-operative BM resection cavities and may support multidisciplinary clinical decision-making when interpreted in the appropriate clinical context. These preliminary findings warrant validation in larger prospective studies.

## 5 Conclusions

Here, we report that TRAMs/CCA may provide complementary information to conventional MRI in the post-operative assessment of BM resection cavities. Previously described imaging appearances after SRS may also be observed before adjuvant treatment and may support multidisciplinary clinical decision-making in selected cases. Further prospective studies are required to validate these findings.

## Data Availability

The original contributions presented in the study are included in the article/Supplementary Material. Further inquiries can be directed to the corresponding author.

## References

[1] Le Rhun E Guckenberger M Smits M Dummer R Bachelot T Sahm F . EANO-ESMO clinical practice guidelines for diagnosis, treatment and follow-up of patients with brain metastasis from solid tumours. Ann Oncol. (2021) 32:1332–47. doi: 10.1016/j.annonc.2021.07.016 34364998

[2] Lescher S Schniewindt S Jurcoane A Senft C Hattingen E . Time window for postoperative reactive enhancement after resection of brain tumors: less than 72 hours. Neurosurg Focus. (2014) 37:E3. doi: 10.3171/2014.9.FOCUS14479 25434388

[3] Jarvis LA Simmons N Bellerive M Erkmen K Eskey CJ Gladstone DJ . Tumor bed dynamics after surgical resection of brain metastases: implications for postoperative radiosurgery. Int J Radiat Oncol Biol Phys. (2012) 84:943–8. doi: 10.1016/j.ijrobp.2012.01.067 22494581

[4] Le Rhun E Dhermain F Vogin G Reyns N Metellus P . Radionecrosis after stereotactic radiotherapy for brain metastases. Expert Rev Neurother. (2016) 16:903–14. doi: 10.1080/14737175.2016.1184572 27177183

[5] Minniti G Clarke E Lanzetta G Osti MF Trasimeni G Bozzao A . Stereotactic radiosurgery for brain metastases: analysis of outcome and risk of brain radionecrosis. Radiother Oncol. (2021) 160:114–21. doi: 10.1186/1748-717x-6-48 21575163 PMC3108308

[6] Milano MT Grimm J Niemierko A Soltys SG Moiseenko V Redmond KJ . Single- and multifraction stereotactic radiosurgery dose/volume tolerances of the brain. Int J Radiat Oncol Biol Phys. (2021) 110:68–86. doi: 10.1016/j.ijrobp.2020.08.013 32921513 PMC9387178

[7] Brun L Dupic G Chassin V Chautard E Moreau J Dedieu V . Hypofractionated stereotactic radiotherapy for large brain metastases: optimizing the dosimetric parameters. Cancer Radiother. (2021) 25:1–7. doi: 10.1016/j.canrad.2020.04.011 33257109

[8] Eitz KA Lo SS Soliman H Sahgal A Theriault A Pinkham MB . Multi-institutional analysis of prognostic factors and outcomes after hypofractionated stereotactic radiotherapy to the resection cavity in patients with brain metastases. JAMA Oncol. (2020) 6:1901–9. doi: 10.1001/jamaoncol.2020.4630 33057566 PMC7563677

[9] Araceli T Haj A Doenitz C Stoerr EM Rosengarth K Schmidt NO . Complete resection of brain metastases – when does it matter? J Neuro Oncol. (2025) 175:1299–309. doi: 10.1007/s11060-025-05193-9 40844730 PMC12511165

[10] Bellomo J Gönel M Sutter J Zeitlberger AM Nikolaeva M Schmidlechner T . Postoperative absence of residual intracranial tumor volume is associated with improved survival and intracranial disease control in non-small cell lung cancer brain metastases. J Neuro Oncol. (2026) 176:131. doi: 10.1007/s11060-025-05387-1 41430011 PMC12722344

[11] Redmond KJ De Salles AAF Fariselli L Levivier M Ma L Paddick I . Stereotactic radiosurgery for postoperative metastatic surgical cavities: a critical review and international stereotactic radiosurgery society (ISRS) practice guidelines. Int J Radiat Oncol Biol Phys. (2021) 111:68–80. doi: 10.1016/j.ijrobp.2021.04.016 33891979

[12] Buszek SM Tran B Long JP Luo D Suki D Li J . Postoperative management of recurrence after radiosurgery and surgical resection for brain metastases and predicting benefit from adjuvant radiation. Pract Radiat Oncol. (2023) 13:e499–503. doi: 10.1016/j.prro.2023.05.010 37295724

[13] Crouzen JA Petoukhova AL Hakstege M van Schaik EEMW Nandoe Tewarie RDS Nabuurs RJA . Patterns of recurrence after postoperative stereotactic radiotherapy for brain metastases. Cancers (Basel). (2025) 17:1557. doi: 10.3390/cancers17091557 40361483 PMC12071874

[14] Martucci M Russo R Giordano C Schiarelli C D'Apolito G Tuzza L . Advanced magnetic resonance imaging in the evaluation of treated glioblastoma: a pictorial essay. Cancers (Basel). (2023) 15:3790. doi: 10.3390/cancers15153790 37568606 PMC10417432

[15] Peker S Samanci Y Aygun MS Yavuz F Erden ME Nokay AE . The use of treatment response assessment maps in discriminating between radiation effect and persistent tumoral lesion in metastatic brain tumors treated with gamma knife radiosurgery. World Neurosurg. (2021) 146:e1134–46. doi: 10.1016/j.wneu.2020.11.114 33253956

[16] Bodensohn R Forbrig R Quach S Reis J Boulesteix AL Mansmann U . MRI-based contrast clearance analysis shows high differentiation accuracy between radiation-induced reactions and progressive disease after cranial radiotherapy. ESMO Open. (2022) 7:100424. doi: 10.1016/j.esmoop.2022.100424 35248822 PMC9058918

[17] Galldiks N Kaufmann TJ Vollmuth P Lohmann P Smits M Veronesi MC . Challenges, limitations, and pitfalls of PET and advanced MRI in patients with brain tumors: a report of the PET/RANO group. Neuro-Oncology. (2024) 26:1181–94. doi: 10.1093/neuonc/noae049 38466087 PMC11226881

[18] Zach L Guez D Last D Daniels D Grober Y Hoffmann C . Delayed contrast extravasation MRI: a new paradigm in neuro-oncology. Neuro Oncol. (2015) 17:457–65. doi: 10.1093/neuonc/nou230 25452395 PMC4483101

[19] Mohamedkhan S Hindocha S de Boisanger J Millard T Welsh L Rich P . Contrast clearance analysis (CCA) to assess viable tumor following stereotactic radiosurgery for NSCLC brain metastasis. Cancers (Basel). (2024) 16:1218. doi: 10.3390/cancers16061218 38539550 PMC10969132

[20] Alkhatatneh H Chen YH Imhoff S Fogel L Yao K Dubin D . Evaluating the diagnostic ability of treatment response assessment maps (TRAMs)/contrast clearance analysis (CCA) in predicting the presence of active brain tumors. Neuroradiol J. (2025) 38:745–51. doi: 10.1177/19714009251324305 40010303 PMC11866328

[21] Cuccarini V Savoldi F Mardor Y Last D Pellegatta S Mazzi F . Response assessment of GBM during immunotherapy by delayed contrast treatment response assessment maps. Front Neurol. (2024) 15:1374737. doi: 10.3389/fneur.2024.1374737 38651109 PMC11033465

[22] Mahajan A Ahmed S McAleer MF Weinberg JS Li J Brown P . Post-operative stereotactic radiosurgery versus observation for completely resected brain metastases: a single-centre, randomised, controlled, phase 3 trial. Lancet Oncol. (2017) 18:1040–8. doi: 10.1016/S1470-2045(17)30414-X 28687375 PMC5560102

[23] Wen PY Van den Bent M Vogelbaum MA Chang SM Cloughesy TF Ellingson BM Weller M . RANO 2.0: update to the response assessment in neuro-oncology criteria for high- and low-grade gliomas in adults. J Clin Oncol. (2023) 41:5187–99. doi: 10.1200/JCO.23.01059 37774317 PMC10860967

